# Implications of Oxidative Stress in the Pathophysiological Pathways of Heart Failure

**DOI:** 10.3390/ijms26115165

**Published:** 2025-05-28

**Authors:** Andrea D’Amato, Claudia Cestiè, Federico Ferranti, Camilla Segato, Silvia Prosperi, Rosanna Germanò, Vincenzo Myftari, Simona Bartimoccia, Valentina Castellani, Roberto Badagliacca, Vittoria Cammisotto, Pasquale Pignatelli, Carmine Dario Vizza, Paolo Severino

**Affiliations:** 1 Department of Clinical, Internal, Anesthesiology and Cardiovascular Sciences, Sapienza University of Rome, Viale del Policlinico 155, 00161 Rome, Italy; claudia.cestie@uniroma1.it (C.C.); federico.ferranti@uniroma1.it (F.F.); segato.1841469@studenti.uniroma1.it (C.S.); silvia.prosperi@uniroma1.it (S.P.); rosanna.germano@uniroma1.it (R.G.); vincenzo.myftari@uniroma1.it (V.M.); roberto.badagliacca@uniroma1.it (R.B.); vittoria.cammisotto@uniroma1.it (V.C.); pasquale.pignatelli@uniroma1.it (P.P.); dario.vizza@uniroma1.it (C.D.V.); paolo.severino@uniroma1.it (P.S.); 2 Department of Medical-Surgical Sciences and Biotechnologies, Sapienza University, 04100 Latina, Italy; simona.bartimoccia@uniroma1.it; 3 Department of Translational and Precision Medicine, Sapienza University of Rome, 00161 Rome, Italy; valentina.castellani@uniroma1.it

**Keywords:** heart failure, pathophysiology, inflammation, guanylate cyclase pathway, mitochondrial dysfunction

## Abstract

Heart failure (HF) is a major socioeconomic problem worldwide, associated with high morbidity and mortality due to several underlying diseases. HF is driven by several closely linked mechanisms whose effects are mutually reinforcing. Some of the signalling pathways involved in the progression of HF may initially be compensatory, such as the renin–angiotensin–aldosterone system (RAAS), whose hyperactivation plays a central role in the progression of HF by promoting fluid retention, inflammation, oxidative stress (OS), and myocardial dysfunction. Fluid retention is also promoted by the action of neprilysin, which contrasts natriuresis and vasodilation. Among the compensatory and subsequently maladaptive systems, chronic hyperactivation of the sympathetic nervous system (SNS) exacerbates maladaptive remodelling and drives the progression of HF. At the molecular level, mitochondrial dysfunction and inflammatory substances are involved in the development of a state of systemic oxidative stress and inflammation. The aim of the following manuscript was to revise the pathophysiology and role of OS in HF, focusing on the current knowledge of the molecular pathways involved.

## 1. Introduction

Heart failure (HF) is a worldwide socioeconomic problem associated with high morbidity and mortality rates [1]. It is a complex and multifaceted syndrome characterised by the inability of the heart to meet the metabolic demands of peripheral organs [1]. Several diseases can lead to HF, including myocardial ischemia, arterial hypertension, valvular heart disease, inflammatory and toxic/metabolic diseases [1,2]. The prevalence and incidence of HF aetiology may vary from country to country [1,2].

Many pathophysiological, molecular and genetic mechanisms are involved in the development and progression of HF. The pathophysiology of HF involves several mechanisms aimed at preserving cardiac function, such as neurohormonal activation, the renin–angiotensin–aldosterone system (RAAS) and sympathetic nervous system (SNS) hyperactivation [1,3,4]. Despite their initial role as compensatory mechanisms, over time they lead to disease progression and determine deterioration of myocardial function, worsening of renal function, fluid overload, and decreased perfusion of end organs in the advanced stages of the disease [3,4].

Oxidative stress (OS), which is associated with several cardiovascular diseases, is determined by an imbalance between the production of reactive oxygen species (ROS) and the natural disposal capacity of antioxidant defence systems [5,6,7,8]. Under physiological conditions, ROS play a central role in many metabolic processes, but under pathological conditions, ROS can trigger multiple reactions leading to damage to proteins, lipids, DNA, and other cellular components, feeding a vicious cycle characterised by increasing inflammation and tissue damage [5,6,7,8]. In HF, ROS may play a central role in pathogenesis, but they may also be a marker of disease severity. Elevated ROS levels are associated with endothelial dysfunction, adverse myocardial remodelling, systemic inflammation, and fibrosis. This implies many hemodynamic changes associated with disease progression, such as decreased cardiac output, increased ventricular filling pressure, and decreased organ perfusion [5,6,7,8].

While in recent years the approach to the treatment of HF has steadily evolved towards patient-tailored management, understanding the molecular pathways underlying the pathophysiological processes in HF may lead to a profound phenotyping of the disease and thus improve patient management [9,10]. The aim of the following manuscript was to review the role of OS in HF, focusing on the current knowledge of the molecular pathways involved. 

This narrative review is based on a comprehensive search of peer-reviewed literature from databases including PubMed, Scopus, and Web of Science, conducted in March 2025. No exclusion for the publishing year was made. Search terms included “heart failure”, “oxidative stress”, “reactive oxygen species”, “neurohormonal activation”, and “antioxidants”. Inclusion was limited to English-language studies with relevance to clinical and molecular mechanisms of HF. Preference was given to recent reviews, landmark trials, and high-quality experimental studies.

## 2. Pathophysiological Mechanisms in Heart Failure and Current Therapeutic Strategies

Many pathophysiological pathways are involved in HF [1,3,4]. These pathways act as compensatory mechanisms in the early stages of the disease, but they create a vicious cycle that leads to cardiorenal and cardiopulmonary syndromes in the advanced stages [1,3,4]. These signalling pathways are of critical importance, as interfering with them in HF may slow disease progression. Furthermore, understanding the importance of these pathophysiologic pathways helps to explain how the use of certain drugs may influence the natural history of the disease. The rationale for the use of RAAS inhibitors (RAASi) such as angiotensin-converting enzyme inhibitors (ACEi), angiotensin receptor blockers (ARBs), sacubitril/valsartan and mineralocorticoid receptor antagonists (MRAs), as well as beta-blockers (BB), sodium glucose co-transporter 2 inhibitors (SGLT2i), and guanylate cyclase stimulators is based on these key pathophysiological principles.

Many ROS are associated with cardiovascular disease representing potential therapeutic targets as well as disease biomarkers [11,12,13]. Among them, hydrogen peroxide (H_2_O_2_), superoxide anion, reactive hydroxyl radical, and peroxynitrite are the main contributors to cell damage in HF. Several enzymes are involved in ROS production. In relation to HF, NADPH oxidase isoforms 1, 2, and 4 (NOX1, NOX2, and NOX 4) are mainly expressed in the myocardium.

### 2.1. Neurohormonal and Neprilysin Pathways

Neurohormonal activation, particularly RAAS hyperactivation, is a key mechanism for the progression of HF [14]. The RAAS regulates the body’s fluid balance, serum electrolyte concentration, and blood pressure. Chronic and acute HF cause hypoperfusion of the end organs. Renal hypoperfusion leads to the release of renin, which promotes the molecular cascade that eventually produces the vasoconstrictor angiotensin II (Ang II), which stimulates aldosterone production and causes sodium and water retention. Chronic hyperactivation of the RAAS stimulates fluid and salt retention, inflammation, and OS. These mechanisms contribute to myocardial dysfunction, increased filling pressure, and adverse myocardial remodelling. The hemodynamic changes and adverse myocardial remodelling induced by the neurohormonal signalling pathway highlight many molecular mechanisms among which OS plays a central role. Reina Couto et al. [15] investigated the relationship between OS and RAAS biomarkers as a function of disease severity and renal function. They demonstrated that patients with chronic HF and impaired renal function have increased intrarenal RAAS activation with higher urinary angiotensinogen and lower serum vitamin D compared to the same patients with normal renal function. In severe chronic HF patients with impaired renal function, urinary isoprostanes are positively associated with circulating angiotensinogen and serum angiotensin-converting enzyme activity [15].

The neprilysin pathway is a central mechanism in the development of HF [16]. Neprilysin is an endopeptidase involved in the degradation of many bioactive molecules such as natriuretic peptides, adrenomedullin, and bradykinin. In HF, chronic salt and fluid overload and subsequent volume and pressure overload of the heart cause cardiomyocytes stretch and release of natriuretic peptides. Natriuretic peptides are involved in many compensatory mechanisms, such as natriuresis, diuresis, and vasodilation. For this reason, inhibition of neprilysin degradation by sacubitril is an important therapeutic mechanism in HF. The neprilysin pathway and OS are closely linked in HF as most of the above bioactive molecules degraded by neprilysin have antioxidant properties.

### 2.2. Adrenergic Hyperactivation 

Hyperactivation of the SNS is one of the most important compensatory and maladaptive mechanisms in HF [17,18,19]. Chronically reduced cardiac output and hypoperfusion of the peripheral organs lead to hyperactivation of the baroreceptors with an increase in sympathetic activity and noradrenaline production. The biological effect unfolds in the blood vessels, the heart muscle, the kidneys, and the central nervous system. Chronic SNS hyperactivation determines the maladaptive remodelling of the myocardium and the deterioration of HF through various mechanisms: (i) increase in heart rate and higher myocardial oxygen consumption through β1-receptors; (ii) diffuse vasoconstriction leading to an increase in afterload and preload with higher myocardial oxygen consumption through α1-receptors; (iii) renin release stimulating the production of angiotensin II and aldosterone with subsequent salt and fluid retention. OS plays a non-negligible role in most of these adverse effects. The release of noradrenaline may directly stimulate NOX enzymes and ROS production in both the heart and the vasculature. There are other mechanisms linking SNS and OS, such as endothelial dysfunction, inflammation, and mitochondrial dysfunction. Hyperactivation of the RAAS and SNS leads to pressure overload and mechanical stretching of the myocardium, which increases the expression of NOX4 [20]. Koba et al. [21] reported that OS is a mediator between RAAS and SNS hyperactivation in chronic HF. Hyperactivation of the RAAS and overproduction of angiotensin II stimulate OS in the peripheral and central nervous system and promote hyperactivation of the SNS in patients with chronic HF.

Disease-modifying therapies for HF may reduce OS, which counteract the deleterious compensatory pathways mentioned above. RAASi may have many potential effects in reducing OS. Sacubitril/valsartan is associated with reductions in NOX2 and 8-isoprostane, NOX, and COX2 inhibition. Circulating aldosterone levels were associated with TIMP-1, ICAM-1, 8-iso-PGF2α, which are systemic markers of OS and inflammation. MRAs reduce NOX expressions, exerting an important antioxidant and antifibrotic effect [20]. ACEi exert an antioxidant effect that reduces inflammation, endothelial dysfunction, and ischemic damage. Especially, molecules such as zofenopril, which contain a sulfhydryl group, may have a complementary effect on free radicals [20]. The combination of RAAS inhibition and the sulfhydryl group is associated with eNOS activation, nitric oxide (NO) production, and reduced lipid peroxidation. Beta-blockers, especially carvedilol, bisoprolol, metoprolol and nebivolol, also improve NO bioavailability and reduce free radical production and lipid cell peroxidation [20].

### 2.3. Cardiometabolic and Cardiorenal Interaction

SGLT2i have revolutionized the treatment of HF. Their mechanism of action goes beyond glucose regulation and affects various metabolic and hemodynamic pathways involved in HF. By inhibiting SGLT2 in the proximal renal tubule, these drugs promote glycosuria and natriuresis, leading to a reduction in plasma volume and preload and an improvement in tubuloglomerular feedback, thereby reducing hyperfiltration and alleviating cardiac stress [22,23,24,25]. In addition, SGLT2i have been shown to improve myocardial energy efficiency by shifting substrate metabolism from glucose to ketone bodies, which serve as a more oxygen-efficient energy source for failing cardiomyocytes [22,23,24,25]. This metabolic adaptation is particularly important in HF with reduced ejection fraction (HFrEF), where mitochondrial dysfunction and OS contribute to disease progression.

In addition, SGLT2i exert direct effects on cardiac myocytes by reducing intracellular sodium and calcium overload through modulation of the Na^+^/H^+^ exchanger, thereby improving contractility and preventing maladaptive remodelling. Savcilioglu et al. [26] demonstrated that treatment with empagliflozin or dapagliflozin in patients with HFrEF significantly improved key echocardiographic parameters, such as left ventricular global longitudinal strain (LV GLS) and left atrial ejection fraction, leading to an improvement in mitochondrial activities, a reduction in OS, and prevention of cardiac fibrosis. Notably, an increase in plasma levels of asprosin, a hormone associated with improved energy metabolism and reduced ROS formation, was observed [26]. These results suggest that SGLT2i may counteract OS in diabetic cardiomyopathy by improving the energy efficiency of the heart muscle. In addition, Hoehlschen et al. [27] emphasised that SGLT2i reduces ROS and reactive nitrogen species (RNS) production by downregulating NOX activity and enhancing endogenous antioxidant defences, thereby mitigating oxidative damage and preserving myocardial integrity. They reported that fibrosis was suppressed by empagliflozin treatment through the inhibition of transforming growth factor β/small mother against decapentaplegic (TGF-β/SMAD) signalling pathway due to the downregulation of TGF-β [27,28]. Nabrdalik-Leśniak et al. [29] analysed both animal models and human tissues and showed that SGLT2i therapy is associated with a reduction in oxidative stress markers, such as lipid peroxidation products and improved antioxidant enzyme activity, which correlate with better cardiac and renal outcomes associated with diabetes-induced OS. The reduction in myocardial and vascular OS biomarkers of lipid peroxidation and nitrosative stress is evident and consistent with the use of empagliflozin or dapagliflozin [30,31]. The cardioprotective effects of SGLT2i are mediated by a complex interplay of improved substrate utilisation, improved mitochondrial function, and attenuation of OS, which together contribute to the amelioration of adverse cardiac remodelling and dysfunction [30,31]. A summary of the principal pharmacological classes used in HF and their associated antioxidant mechanisms is provided in Table 1.

## 3. Inflammation and Oxidative Stress in Heart Failure

OS and inflammation are involved in the development and progression of both acute and chronic HF [6,32]. Two major signalling pathways, the nuclear factor kappa-light-chain-enhancer of activated B cells (NF-κB) pathway and the mitogen-activated protein (MAP) kinase pathway, are involved in mediating this process. 

ROS can be produced via the transport chain in the mitochondria by ionizing radiation or by reactions that generate superoxide anions [33]. Depending on the cellular ROS level, different reactions can be triggered. 

Among the enzymatic sources of ROS, NADPH oxidases—particularly NOX2 and NOX4—are prominent in the myocardium. These isoforms have distinct regulatory mechanisms and exert differential effects on cardiac physiology and pathology [34] NOX2, in particular, plays a key role in maladaptive remodelling after myocardial infarction [35]. Experimental studies have shown that NOX2 activity in cardiomyocytes, rather than in endothelial cells, drives post-infarction left ventricular dilation and systolic dysfunction [35]. Genetic deletion of NOX2 in murine models attenuates remodelling and preserves cardiac function [35]. 

Beyond ischemic injury, NOX2-derived ROS are also implicated in atrial remodelling and the development of atrial fibrillation (AF), a common comorbidity in HF that further exacerbates hemodynamic compromise. In human atrial myocytes, NOX2 is a major source of superoxide, with elevated NOX2 activity (measured via soluble NOX2-derivated peptide) observed in patients with paroxysmal, persistent, and permanent AF [36]. This marker correlates with systemic oxidative stress indicators such as urinary isoprostanes and has been associated with increased AF risk in inflammatory conditions like community-acquired pneumonia [37].

The growing body of evidence supports NOX2 as a significant contributor to cardiac oxidative damage in HF. Accordingly, soluble NOX2-derivated peptide (sNOX2-dp) may serve as a promising biomarker for identifying patients at risk of adverse remodelling and arrhythmic complications, as well as for monitoring disease progression and therapeutic response.

### 3.1. Nuclear Factor Kappa-Light-Chain-Enhancer of Activated B Cells (Nf-κB) Signalling

**Figure 1:**
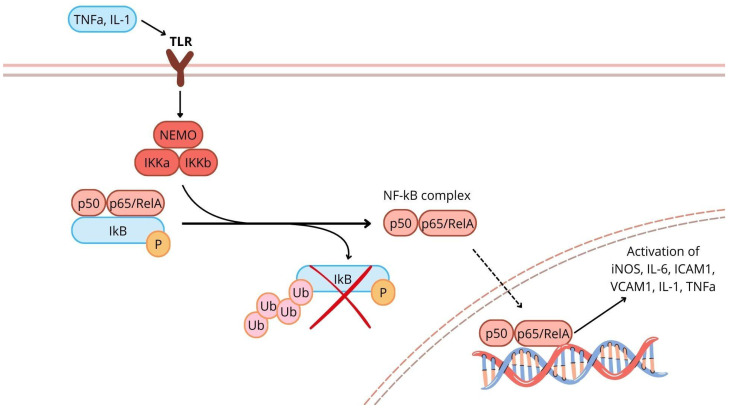
NF-κB signalling pathway leading to the expression of genes encoding inducible nitric oxide synthase (iNOS), interleukin-6 and adhesion molecules (i.e., ICAM-1 and VCAM-1), immune receptors and cytokines (i.e., interleukin-1 and TNF). TNF-α: tumour necrosis factor α; IL-1: interleukin-1; TLR: toll-like receptor; NEMO: NF-κB essential modulator; IkB: inhibitor kappa B; IKK: IkB-kinases; NF-κB: nuclear factor kappa-light-chain-enhancer of activated B cells; iNOS: inducible nitric oxide synthase; ICAM1: intercellular adhesion molecule-1; VCAM1: vascular cell adhesion molecule-1; P: phosphate.

A high OS status leads to apoptosis or necrosis, while an intermediate level can trigger inflammatory responses through the activation of molecular signalling pathways such as NF-κB [33]. NF-κB is a transcription factor that modulates the gene expression of various molecules involved in inflammation, immunity, apoptosis, and cell proliferation. The NF-κB family consists of five proteins (p50, p52, p65/RelA, c-Rel, and RelB) that form homo- or heterodimers. The most common active form is the heterodimer consisting of p50-RelA or p52-RelA [38]. In the resting state, the heterodimer is inactivated by binding to inhibitory proteins called inhibitor kappa B (IkBs) and is located in the cellular cytoplasm. These inhibitory proteins are equipped with two N-terminal serine residues that are targeted for phosphorylation by the IkB kinases (IKK) complex, as a result of various stimuli. The IKK complex comprises the scaffold protein NF-κB essential modulator (NEMO), IKKα, and IKKβ. The degradation of IkBs determines the activation of the NF-κB complex, which can thus enter the cell nucleus, bind to promoter regions, and initiate the transcription of specific genes, leading mainly to the production of inflammatory proteins [38]. In addition to the classical activation, an alternative, NEMO-independent pathway has also been discovered. This pathway is mainly involved in the development of lymphoid organs and in the production of homeostasis proteins [33]. OS may therefore be involved in NF-κB activation via the classical pathway. The primary ROS investigated in the activation of NF-κB is H_2_O_2_. This ROS is generated by the dismutation reaction with the superoxide anion, which can be spontaneous or catalysed by superoxide dismutase. Of particular interest is the remarkable cell type specificity of NF-κB activation by H_2_O_2_ [33]. Once activated, NF-κB is involved in the expression of genes encoding inducible nitric oxide synthase (iNOS), interleukin-6 (IL-6) and adhesion molecules (e.g., ICAM-1 and VCAM-1), immunoreceptors, and cytokines (e.g., IL-1 and TNF). The latter, once produced, can themselves activate NF-κB and thus maintain the inflammation process itself [38]. Of the TNF family, TNF-α appears to be associated with the development of HF, as it has a negative inotropic effect. Indeed, TNF-α appears to induce a change in intracellular calcium homeostasis, leading to contractile dysfunction [39]. In addition, TNFα may act directly by altering the activity of the β-adrenergic receptor (βAR). Chronic βAR activation may further increase TNFα expression and thus contribute to the self-perpetuation of inflammation and chronic myocardial dysfunction [40]. The NF-κB pathway is illustrated in Figure 1.

### 3.2. Mitogen-Activated Protein (MAP) Kinase Pathway

The MAPK signalling pathway is composed by a family of ubiquitous serine and threonine protein kinases which can be activated by various stress signals, including inflammatory cytokines and OS. Three MAPKs, in particular, are activated by stress and appear to be involved in the development and progression of HF. These three MAPKs are as follows: extracellular signal-regulated kinases (ERKs), p38 kinase, and c-jun N-terminal protein kinases (JNKs) [41].

On cardiac fibroblasts, the ERK1/2 signalling pathway is activated by either receptor tyrosine kinases (RKTs) or G protein-coupled receptors (GPCRs). The RKT activators are growth factors that appear to be produced at high levels in advanced stages of cardiovascular disease, hypoxia, and stress associated with inflammation. In another way, GPCRs can be activated by Ang II, levels of which appear to be higher in HF patients [42]. Once activated, ERK1/2 stimulates transcription factors such as c-myc, c-jun, and c-fos [41]. Activation of these signalling pathways increases cardiac fibroblast activity [42].

Similarly, the JNK signalling pathway can be activated by TNF, growth factors, and stress responses. The receptor involved in the activation of this pathway is TLR4, which is mainly activated by Ang II and advanced glycation end-products (AGE). Once activated, JNK migrates from the cytoplasm to the nucleus and increases the expression of c-Jun [42].

Once expressed, c-jun and c-fos combine to form a heterodimer that forms activator protein 1 (AP-1). In cardiac fibroblasts, AP-1 (c-Jun/c-Fos) can translocate through the nuclear pore into the nucleus after activation by Ang II. There it binds to the AP-1 binding site in the promoter regions of genes associated with cell proliferation and collagen maturation, thereby enhancing the transcriptional activity of these genes. The increased expression of the AP-1 targeted genes, cyclin D1 (CCND1) and lysyl oxidase (LOX), promotes the proliferation, migration, and differentiation of cardiac fibroblasts. This in turn increases the production of collagen type I and type III (COL I/III) in cardiac tissue and promotes its cross-linking with elastin, which ultimately leads to an accumulation of mature collagen in the extracellular matrix and accelerates the development of myocardial fibrosis [42]. It has been shown that AP-1 and NF-κB are strongly activated during left ventricular remodelling in congestive HF [43]. 

Stress and proinflammatory cytokines, such as IL-1 and TNF, regulate the activity of the p38 kinase, which belongs to the MAPK family. The biological activity of p38 kinase is not yet fully understood, but it appears to be involved in the biosynthesis of proinflammatory cytokines [41]. The p38 kinase has four isoforms (p38α, p38β, p38γ, and p38δ), of which the first two isoforms are found in the myocardium [41]. p38α is activated during myocardial ischemia, while p38β is inhibited. This emphasises the possible protective role of this isoform of p38β [44,45].

With respect to the HF stage, p38 appears to be activated and to have a negative inotropic effect. This effect appears to be due to changes in the response of myofilaments to intracellular calcium concentration. Furthermore, p38 appears to enhance the negative effect of TNFα on contractility [46].

Recent evidence suggests a potential role for brain-derived neurotrophic factor (BDNF) in the pathophysiology of cardiovascular disease, including HF. BDNF is synthesized not only in neurons but also in cardiomyocytes, endothelial cells, platelets, and vascular smooth muscle cells, where it modulates cardiac development, function, and response to injury through its high-affinity receptor TrkB [47,48]. Alterations in the BDNF-TrkB signalling pathway have been linked to impaired cardiac contraction and relaxation, increased OS and inflammation, and adverse myocardial remodelling [47,48]. In patients with systolic HF, lower circulating BDNF levels are independently associated with more severe structural cardiac changes, worse echocardiographic parameters, higher N-terminal pro B-type Natriuretic Peptide (NT-proBNP) levels, and worse clinical outcomes, supporting its utility as a biomarker of disease severity [47,48]. Moreover, in patients with acute coronary syndromes, higher BDNF levels correlate with markers of coronary inflammation such as macrophage infiltrates and are inversely associated with the presence of healed plaques, suggesting a role for BDNF in thrombo-inflammatory activation and plaque destabilization [47,48]. Together, these findings support the hypothesis that BDNF is involved in the interplay between OS, neurohormonal dysregulation, and cardiac remodelling in HF. 

In the light of the evidence presented above, the NF-κB and MAP kinase signalling pathways appear to be activated under OS and inflammatory conditions, in part through interactions between them, thereby contributing to the progression of HF. Alongside these classical pathways, recent findings suggest that dysregulation of BDNF signalling may further amplify oxidative damage, inflammation, and maladaptive cardiac remodelling. Together, these interconnected mechanisms highlight novel potential targets for future therapeutic strategies in HF.

## 4. Endothelial Dysfunction and the Guanylate Cyclase Pathway in Heart Failure

**Figure 2:**
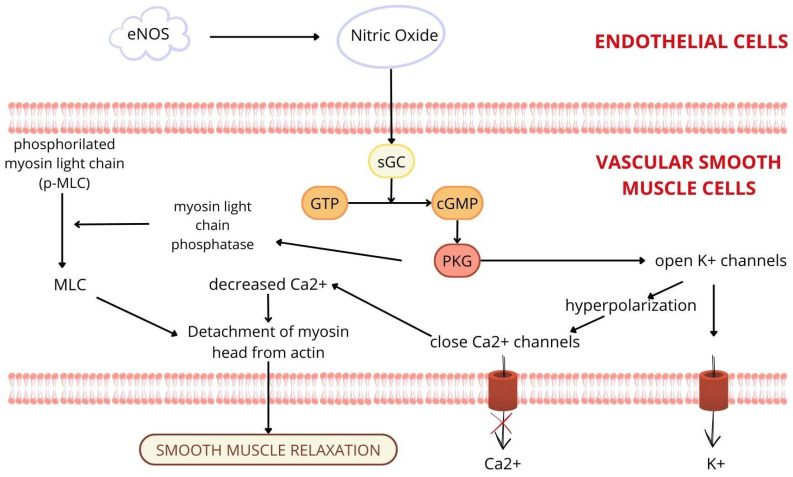
Nitric oxide is synthesised by endothelial nitric oxide synthase (eNOS) in the endothelial cells and diffuses into vascular smooth muscle cells, where it stimulates the soluble guanylate cyclase (sGC) pathway, which causes vasodilation and smooth muscle relaxation. eNOS: endothelial nitric oxide synthase; sGC: soluble guanylate cyclase; GTP: guanosine triphosphate; cGMP: cyclic guanosine monophosphate; 5’ GMP: 5’ guanosine monophosphate; PKG: protein kinase G; MLC: myosin light chain.

Under physiological conditions, the vascular endothelium plays a crucial role in the regulation of vascular tone, primarily through the production and release of NO. NO is synthesised from L-arginine by eNOS. Endothelial cells are stimulated to release NO by mechanical forces, such as shear stress and signals from acetylcholine, adenosine diphosphate, bradykinin, thrombin, and serotonin. After synthesis, NO diffuses into vascular smooth muscle cells and activates soluble guanylate cyclase (sGC), increasing intracellular cyclic guanosine monophosphate (cGMP), which causes vasodilation and smooth muscle relaxation (Figure 2). In addition, the endothelium has antiproliferative and anti-inflammatory effects and regulates fibrinolysis and coagulation pathways through the balanced production of anticoagulant factors (e.g., tissue plasminogen activator, thrombomodulin) and procoagulant factors (e.g., tissue factor, von Willebrand factor), which together maintain vascular haemostasis. These functions play a crucial role in maintaining vascular integrity and homeostasis [49,50].

In HF, however, endothelial dysfunction results from an imbalance in endothelial regulatory mechanisms, which is significantly influenced by an inflammatory and OS environment. The inflammatory state characteristic of HF significantly increases OS and ROS production, primarily through activation of NOX enzymes, xanthine oxidase, and dysfunction of mitochondrial electron transport chain (ETC) [49,50,51,52,53]. Specially, NOX enzymes and xanthine oxidase contribute to the formation of superoxide radicals. These superoxide radicals react rapidly with NO to form peroxynitrite, which severely limits NO bioavailability and exacerbates endothelial dysfunction. In addition, increased levels of circulating cytokines, particularly TNF-α, have been observed in HF, leading to downregulation of constitutive eNOS expression in the endothelium and increased endothelial apoptosis. This combination of decreased eNOS expression, increased superoxide generation, and endothelial apoptosis leads to a further reduction in NO bioavailability, which exacerbates endothelial dysfunction and impairs vasodilation [49,50,51,52,53].

The manifestations of endothelial dysfunction differ markedly between the various HF phenotypes, particularly HFrEF and HF with preserved ejection fraction (HFpEF) [51,52,54]. In HFpEF, endothelial dysfunction is primarily characterised by inflammation-induced impairment of coronary microvascular endothelial function. This dysfunction results from systemic inflammation associated with common comorbidities such as arterial hypertension, diabetes, obesity, and chronic kidney disease. The inflammatory milieu leads to decreased NO bioavailability and impaired cGMP–protein kinase G (PKG) signalling, which promotes cardiomyocyte hypertrophy, increased myocardial stiffness, and increased interstitial fibrosis mediated by TGF-β via hypophosphorylation of titin. In contrast, endothelial dysfunction in HFrEF is typically caused by damage to cardiomyocytes due to OS resulting from ischemia, infarction, or direct myocardial injury. This OS primarily results in loss of cardiomyocytes, eccentric remodelling of the heart, and fibrosis replacement that further impairs cardiac function. These different pathological mechanisms emphasise the importance of phenotype-specific therapeutic strategies targeting endothelial dysfunction [51,52,54].

Given the central role of the NO–sGC pathway in endothelial function, pharmacological strategies targeting this pathway are of great importance for therapeutic interventions. Vericiguat, a novel oral sGC stimulator, increases the sensitivity of sGC to endogenous NO and enhances cGMP production, regardless of NO availability. The VICTORIA study [55], a randomized, placebo-controlled phase 3 clinical trial involving 5050 patients with chronic HFrEF, showed a significant reduction in the composite primary endpoint of cardiovascular death or first hospitalization for HF in patients receiving vericiguat compared to placebo. These results confirm the positive effect of vericiguat in improving clinical outcomes in HFrEF following the potential molecular benefits of this drug. However, in the HFpEF phenotype, the use of this drug has shown limited efficacy. In the VITALITY-HFpEF trial [56], vericiguat did not significantly improve patient-reported outcomes as measured by the Kansas City Cardiomyopathy Questionnaire or exercise capacity as measured by the 6-minute walk test compared to placebo. These contrasting results emphasize the complexity of treating endothelial dysfunction in different HF phenotypes and highlight the need for tailored therapeutic approaches [54,55,56,57].

In summary, endothelial dysfunction is an important therapeutic target in the treatment of HF. Future research should aim to clarify the different pathophysiologic mechanisms between HFrEF and HFpEF and promote phenotype-specific treatments. While the improvement of NO–sGC–cGMP metabolism in HFrEF has been promising, as shown by the efficacy of vericiguat in the VICTORIA trial [55], results in HFpEF, such as those of the VITALITY-HFpEF trial [56], have been less encouraging. Tailored therapies targeting phenotype-specific mechanisms of endothelial dysfunction, including inflammation and OS, are essential for improving clinical outcomes in HF patients [54,55,56,57].

## 5. Mitochondrial Dysfunction and Oxidative Stress in Heart Failure

Mitochondria play an essential role in normal cardiac physiology, primarily through energy production by oxidative phosphorylation. They produce approximately 95% of the adenosine triphosphate (ATP) required to meet the extensive energy demands of the heart [58,59]. This process involves electron transfer through the ETC complexes (I–IV) within the inner mitochondrial membrane, where the proton gradient generated drives ATP synthesis by ATP synthase [46,56]. Mitochondria are also involved in calcium homeostasis, apoptosis regulation, and redox signalling, processes that are critical for cardiomyocyte viability and maintenance of cardiac function [59,60]. Approximately 30–40% of the volume of cardiomyocytes is occupied by mitochondria, reflecting the high energy requirements of the organ [59].

**Figure 3:**
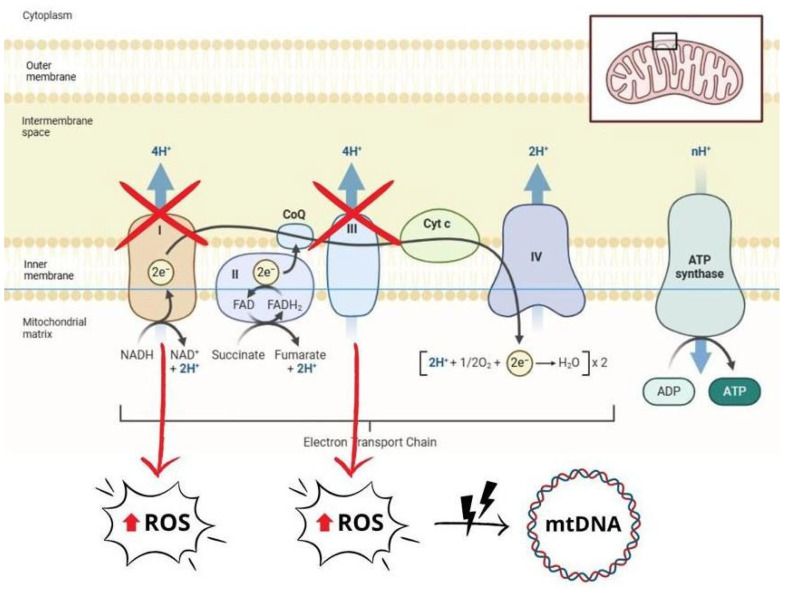
Schematic representation of mitochondrial dysfunction, which contributes to increased production of reactive oxygen species (ROS). Impaired electron transport chain (ETC) activity, particularly in complexes I and III, leads to electron loss and a subsequent increase in ROS levels. Excessive ROS cause oxidative damage that affects mitochondrial DNA (mtDNA), proteins, and lipids, exacerbating mitochondrial dysfunction and cell damage. ROS: reactive oxygen species; mtDNA: mitochondrial DNA; ATP: adenosine triphosphate; ADP: adenosine diphosphate; NADH: nicotinamide adenine dinucleotide (reduced form); NAD+: nicotinamide adenine dinucleotide (oxidized form); FADH₂: flavin adenine dinucleotide (reduced form); FAD: flavin adenine dinucleotide (oxidized form); CoQ: coenzyme Q (ubiquinone); Cyt c: cytochrome c.

In HF, mitochondrial dysfunction significantly impairs myocardial bioenergetics and contributes to disease progression [61]. Several dysfunctional mechanisms have been described, including impaired oxidative phosphorylation, increased ROS generation, abnormal mitochondrial dynamics, mitochondrial DNA (mtDNA) damage, and alterations in mitochondrial biogenesis and mitophagy [58,59,62]. In particular, decreased activities of ETC complexes I and III lead to decreased ATP generation and increased electron loss, resulting in excessive ROS production [58,59,63]. ROS excess causes oxidative damage to mtDNA, proteins and lipids, leading to mitochondrial dysfunction and energy depletion. This damage activates cardiomyocyte death pathways, including apoptosis and regulated necrosis, by opening the mitochondrial permeability transition pore (mPTP) and permeabilizing the mitochondrial outer membrane [5,61,63,64]. Notably, mtDNA is highly susceptible to ROS-induced damage. In contrast to nuclear DNA, mtDNA lacks histone-based chromatin organization, making it more susceptible to OS. In addition, the reduced efficiency of mtDNA repair systems and the accumulation of ROS in the mitochondrial matrix further exacerbate the damage [63,65]. These processes initiate a deleterious feedback loop in which mitochondrial dysfunction exacerbates OS, leading to further mtDNA damage, impaired energy production, and increased apoptosis. This cycle ultimately drives myocardial remodelling, fibrosis, and clinical progression of HF through persistent OS, calcium dysregulation, and inflammatory signals. [5,58,59,64] (Figure 3).

While mitochondrial dysfunction is a common feature of all HF phenotypes, subtle differences between HFrEF and HFpEF have been highlighted [66,67]. In HFpEF, mitochondrial impairment contributes to diastolic dysfunction, primarily through OS, inflammation, and calcium dysregulation. Elevated ROS levels contribute to endothelial dysfunction and microvascular rarefaction, which impairs myocardial perfusion and exacerbates diastolic dysfunction. In addition, mishandling of mitochondrial calcium contributes to delayed relaxation of cardiomyocytes, while chronic inflammation, often associated with systemic comorbidities, such as diabetes and arterial hypertension, exacerbates OS and metabolic abnormalities, further impairing mitochondrial function [66]. Conversely, mitochondrial dysfunction in HFrEF significantly impairs energy production, leading to ATP depletion and the inability to meet the contractile demands of the heart. The failing mitochondria produce excessive ROS, further damage of mtDNA, proteins, and lipids, exacerbating cellular dysfunction. In contrast to HFpEF, where OS primarily affects endothelial cells, ROS accumulation in HFrEF directly damages cardiomyocytes by triggering mPTP opening, cytochrome c release, and caspase-dependent apoptosis. This progressive loss of cardiomyocytes leads to fibrosis and remodelling and exacerbates contraction weakness [66,67,68,69].

Given the central role that mitochondrial dysfunction plays in the pathophysiology of HF, targeting the mitochondria is an attractive therapeutic option. Current approaches include mitochondria-targeted antioxidants (e.g., MitoQ, coenzyme Q10), metabolic modulators, such as SGLT2i that improve mitochondrial bioenergetics and substrate utilization efficiency, and agents aimed at modulating mitochondrial dynamics and improving mitochondrial quality control through biogenesis or mitophagy. Clinical evidence from the Q-SYMBIO trial [70] showed that supplementation with coenzyme Q10 (CoQ10) can reduce mortality by attenuating OS and improving mitochondrial function. This emphasizes the therapeutic potential of direct treatment of mitochondrial dysfunction [60,68,69]. Similarly, SGLT2i have shown significant clinical benefit by reducing mitochondrial OS, improving myocardial substrate metabolism, and reducing maladaptive remodelling [24,62]. Despite the encouraging preclinical results, it currently remains a challenge to translate these therapies into widespread clinical use. 

A summary of the role of endothelial and mitochondrial dysfunction in HFpEF and HFrEF is provided in Table 2.

## 6. Effect of Antioxidants on Heart Failure

Given the central role of oxidative stress in the pathophysiology of HF, several antioxidant therapies have been investigated in clinical trials with the aim of improving outcomes in affected patients. Among these, CoQ10 has received particular attention.

In the Q-SYMBIO trial [70], a multicentre, randomised, double-blind, placebo-controlled trial, 420 patients with chronic HF received either 100 mg of CoQ10 three times a day or placebo in addition to standard therapy for a period of two years. Long-term CoQ10 supplementation significantly reduced major adverse cardiovascular events, including cardiovascular and all-cause mortality [70]. These positive findings were further supported by a meta-analysis focusing on patients with HFrEF, which confirmed that adding CoQ10 to conventional therapy improved clinical prognosis [71].

Conversely, less favourable results were obtained with the use of vitamin E as an antioxidant intervention in HF. In fact, the large-scale HOPE trial [72] evaluated long-term supplementation with 400 IU of vitamin E daily in individuals at high cardiovascular risk. The results showed that long-term vitamin E supplementation does not prevent major cardiovascular events and may increase the risk for HF and hospitalization for HF [72]. Similarly, another randomised controlled trial by Keith et al. [73] evaluated vitamin E supplementation (800 IU daily for 12 weeks) in patients with advanced HF to assess its effect on markers of OS. Although supplementation increased plasma α-tocopherol levels, it failed to significantly affect marker of OS or improvement of patient quality of life [73]. Larger evaluations of the use of antioxidant vitamins have largely confirmed the lack of clinical benefit observed in individual trials. The results of a meta-analysis of the effects of vitamin E, vitamin C, and β-carotene showed no significant effect on the incidence of cardiovascular events, myocardial infarction, stroke or total death, and cardiac death [74]. 

Omega-3 polyunsaturated fatty acids (n-3 PUFAs) have shown more encouraging results. The GISSI-HF trial [75] demonstrated that 1 g/day of n-3 PUFAs reduced all-cause mortality and cardiovascular hospitalizations in patients with chronic HF, with a favourable safety profile. Beyond clinical outcomes, experimental studies have shown that n-3 PUFAs exert multiple cardioprotective effects, including anti-inflammatory, antioxidant, and anti-fibrotic effects. They improve endothelial function, modulate autonomic tone, and protect against adverse post-infarction remodelling. These mechanisms translate into improved left ventricular ejection fraction, diastolic function, and peak oxygen consumption, along with reductions in brain natriuretic peptide, hsCRP, and norepinephrine levels, with positive effects on cardiac hemodynamic [76]. Additionally, a recent meta-analysis [77] confirmed that only high-dose (≥2000 mg/day) and long-term (≥1 year) supplementation significantly improves systolic function and volume of oxygen (VO_2_) peak, further supporting the dose- and time-dependent benefits of n-3 PUFAs in HF management. 

Although antioxidant therapies like coQ10 to omega-3 PUFAs have shown encouraging results in their relative clinical studies, their integration into routine HF management remains limited. 

The 2022 AHA/ACC/HFSA Guidelines for HF [78] recognize omega-3 PUFAs as potential adjunctive therapies, citing evidence from large, randomized trials that demonstrate modest reductions in cardiovascular mortality and hospitalizations, particularly in patients with chronic HFrEF. In contrast, coQ10 is included among the therapies not recommended, due to the absence of consistent symptomatic benefit. While some studies have reported potential prognostic advantages, these findings remain insufficient [78]. In contrast, the 2021 ESC Guidelines for HF [1], including the 2023 focused update [79], make no mention of either coQ10 or omega-3 PUFAs in the HF pharmacological treatment.

Both the AHA/ACC/HFSA and ESC Guidelines for HF [1,78,79] adopt a cautious position toward emerging antioxidant therapies in HF. This conservative approach likely reflects several factors, including the absence of large, high-quality randomized controlled trials, heterogeneity in dosing and formulations across studies, and in some cases the lack of available evidence at the time of guidelines publication, with certain relevant findings emerging only more recently.

At present, antioxidant strategies remain outside the core recommendations for HF treatment, and their clinical utility is limited by the absence of strong support from major guidelines.

In conclusion, while current evidence is not yet sufficient to support widespread implementation of antioxidant therapies, ongoing research may shift this paradigm. With future high-quality trials, these therapies may potentially shift from adjunctive options to important elements in the management of HF.

## 7. New Perspectives: Biomarkers of Oxidative Stress in Heart Failure

A biomarker is defined as a measurable characteristic that serves as an indicator of pathogenic processes or as a tool for monitoring patients undergoing pharmacological therapy [80]. It is well known that patients with HF have a highly fragile balance, which can be disrupted by minor factors (such as infections, atrial fibrillation, etc.), often leading to frequent hospitalizations. In this context, biomarkers may play a pivotal role in guiding and optimizing therapeutic decisions, thereby helping to prevent severe clinical manifestations of the disease.

Based on the key role of OS in the pathophysiology of HF, it may be understandable that the direct measurement of ROS could be useful for assessing disease severity and progression. However, ROS are difficult to measure directly due to their intrinsic instability and their widespread distribution throughout the body, which makes them poorly accessible. Therefore, indirect markers of OS are more commonly employed, and, among them, certain ones exhibit clinical significance. 

For instance, by assessing NOX activity through an antibody that binds to the extracellular domain of NOX2, it was observed that this antibody also bound to a peptide, which was therefore defined as the sNOX2-dp. As NOX appears to be the primary contributor to ROS production in pressure overload-induced left ventricular hypertrophy, NOX2-dp may be of particular interest. However, findings about the stability, metabolism, and clearance of this peptide are still insufficient and further studies are needed [81].

Other markers of OS that may be of interest in clinical practice are F2-isoprostanes. They are produced, in the presence of ROS, from the peroxidation of arachidonic acid, and their stability, sensitivity to OS exposure, and easy collection by urine and serum sample make them a promising biomarker [81]. Many studies have focused on the correlation between F2-isoprostanes and HF diagnosis and prognosis, demonstrating their promising potential [82,83]. However, further long-term studies are required to determine their usefulness and whether there are differential expressions in HFrEF and HFpEF [81]. 

Another OS clinically useful marker can be identified in AGEs that result from the oxidation and nonenzymatic glycation of proteins. At the molecular lever, AGEs are thought to bind among matrix proteins reducing their flexibility of affecting diastolic function. Moreover, the activation of their receptor (RAGE) promotes fibrosis by enhancing the expression of TGF-β and diminishes contractility by disrupting calcium metabolism in cardiomyocytes. These markers may have an important role in the management of both HFpEF and HFrEF patients. Especially, RAGE seems to be more expressed in HFpEF while AGEs seem to be predominant in HFrEF [81].

In conclusion, numerous indirect markers of OS have been identified, some of which are easy to sample and assess, and which could have a potential role in the management of patients with HF and further personalization of therapy. However, further studies, including long-term studies, are required before these markers can be incorporated into clinical practice. 

## 8. Conclusions

Due to the increasing ability to treat cardiovascular disease associated with an aging population, HF has become an increasingly prevalent syndrome that represents a high socioeconomic burden. As a result, it has become an area of intense research aimed at identifying the most effective therapies to improve both the prognosis and quality of life of patients. In this context, personalized therapy, which takes a holistic view of the patient, is playing an increasingly important role. Key areas of interest include OS in relation to mitochondrial dysfunction, inflammation, endothelial dysfunction, and various molecular signalling pathways involved in HF. OS is a complex network consisting of many molecules and signalling pathways involved in the development and progression of HF. For this reason, the molecules involved in OS could represent new biomarkers for the disease in view of an even more personalized approach, allowing an in-depth characterization of HF.

In summary, thorough understanding of the underlying pathophysiology and identification of the various etiological mechanisms underlying the condition of HF are therefore of paramount importance [84]. This applies not only to the rationale of current pharmacological strategies [85,86] included in clinical guidelines, but also to the identification of new biomarkers and new pharmacological targets that could further improve the prognosis and quality of life of patients.

In this context, antioxidant therapies remain a potential area of interest, despite mixed clinical trial results. As oxidative stress plays a pivotal role in the progression of HF, antioxidants may offer an interesting therapeutic approach, in addition to standard therapy or in combination with new strategies targeting molecular pathways involved in HF progression. However, further studies are needed to establish their role and therapeutic potential in the management of HF.

## Figures and Tables

**Table 1:** Summary of major pharmacological classes used in heart failure with documented antioxidant effects. The table outlines key mechanisms of action and molecular targets for each therapeutic group.

Drug Class	Antioxidant Mechanism	Molecular Target
RAAS Inhibitors (ACEi/ARBs/MRAs)	Reduce NOX expression, lower oxidative enzymes, anti-inflammatory	NOX enzymes, Aldosterone receptors, ACE/AT1R
Sacubitril/Valsartan	Inhibits NOX2, reduces 8-isoprostane, enhances NO signalling	NOX2, neprilysin pathway, COX2
Beta-Blockers	Increase NO bioavailability, reduce lipid peroxidation	β-adrenergic receptors, endothelial NO synthase
SGLT2 Inhibitors	Decrease ROS/RNS, shift metabolism to ketone bodies, inhibit NOX	Na⁺/H⁺ exchanger, mitochondrial enzymes, NOX
Coenzyme Q10	Improves mitochondrial function, reduces lipid peroxidation and apoptosis	Mitochondrial ETC complexes I and III
Omega-3 PUFAs	Membrane stabilization, inhibit fibroblast activation, reduce ROS/inflammation	GPR120, NRF2, mitochondrial membranes
Guanylate Cyclase Stimulators (e.g., Vericiguat)	Improve NO-cGMP pathway, reduce endothelial ROS, enhance vasodilation	sGC, NO signaling

RAAS: renin–angiotensin–aldosterone system; ACEi: angiotensin-converting enzyme inhibitor; ARBs: angiotensin receptor blockers; MRAs: mineralocorticoid receptor antagonists; SGLT2i: sodium–glucose cotransporter 2 inhibitors; PUFAs: polyunsaturated fatty acids; NOX: NADPH oxidase; ACE/AT1R: angiotensin converting enzyme/angiotensin II type 1 receptor; NO: nitric oxide; COX: cyclooxygenase; ROS: reactive oxygen species; RNS: reactive nitrogen species; ETC: electron transport chain; GPR120: G protein-coupled receptor 120; NRF2: nuclear factor erythroid 2-related factor 2; NO-cGMP: nitric oxide/cyclic guanosine monophosphate; sGC: soluble guanylate cyclase.

**Table 2:** Different molecular pathways and subsequent cellular modifications in HFpEF and HFrEF. HFrEF: heart failure with reduced ejection fraction.

		HFpEF	HFrEF
Endothelial dysfunction	***Molecular*** ***Pathway*** ***Cellular modifications***	Decreased NO bioavailability + Impaired cGMP–PKG signalling Cardiomyocyte hypertrophy Increased myocardial stiffness Increased interstitial fibrosis	Oxidative stress resulting from ischemia, infarction or direct myocardial injury Loss of cardiomyocytes Eccentric remodelling of the heart Fibrosis replacement
Mitochondrial dysfunction	***Molecular*** ***Pathway*** ***Cellular modifications***	Elevated ROS level + Mishandling of mitochondrial calcium Impaired myocardial perfusion Exacerbated diastolic dysfunction Delayed relaxation of cardiomyocytes	ATP depletion + Augmented caspase-dependent apoptosis due to ROS accumulation Inability to meet the contractile demands of the heart Loss of cardiomyocytes Fibrosis and remodelling Contraction weakness

HFpEF: heart failure with preserved ejection fraction; NO: nitric oxide; cGMP-PKG: cyclic guanosine monophosphate/protein kinase G; ROS: reactive oxygen species; ATP: adenosine triphosphate.
